# Retrospective investigation of organization and examination results of the state examination in restorative dentistry, endodontology and periodontology under simulated conditions in times of Covid-19 compared to standard conditions when treating patients

**DOI:** 10.3205/zma001380

**Published:** 2020-12-03

**Authors:** Michael J. Wicht, Karolin Höfer, Sonja H. M. Derman, M. J. Noack, A. Greta Barbe

**Affiliations:** 1 Uniklinik Köln, Zentrum für Zahn-, Mund und Kieferheilkunde, Poliklinik für Zahnerhaltung und Parodontologie, Cologne, Germany

**Keywords:** state examination, Covid-19, dentistry, simulated treatment

## Abstract

**Objective: **Primary outcome of this retrospective study was the comparison of state examination results under simulated treatment conditions in times of Covid-19 versus patient treatment under non-pandemic conditions. Additionally, correlation analysis was performed between students’ self- and examiners’ assessment of the treatment results.

**Methods: **Within 4 hours, 22 examinees each had to place a multi-surface adhesive anterior and posterior restoration, performed an endodontic treatment on a maxillary premolar and a periodontal debridement of one quadrant. All treatments were performed on a model fixed in a phantom head. Compliance with the prescribed hygiene and social distancing guidelines and self-assessment of the practical performance was part of the practical examination as well. One examiner per examination part evaluated anonymously the final results. The historical control was based on the exam results of a cohort from 2019. Mean values (standard deviation), non-parametric correlations (Spearman's Rho) and group comparisons (Mann-Whitney) were calculated for statistical analysis.

**Results:** Examination results under simulated treatment conditions were significantly worse (p<0.05) than in the cohort that took their state exam in patients, with exception of the endodontic partial exam. The overall scores in restorative dentistry and periodontology of both groups, which include a structured theoretical examination, did not differ. The majority of the candidates rated their performance worse than the examiners, and there was no correlation between self- and third-party assessment.

**Conclusion:** In the comparison of two years, a simulated practical examination without patients in restorative dentistry, endodontics and periodontology resulted in matchable results compared with an examination on patients. Equal conditions for the candidates resulting in better comparability and avoidance of ethical dilemmas of patient treatment under examination conditions could also be arguments towards a state examination under phantom conditions in the future.

## Zusammenfassung

**Zielsetzung:** Primärer Outcome der retrospektiven Studie war ein Vergleich der Prüfungsergebnisse im Staatsexamen unter simulierten Behandlungsbedingungen am Phantom in Zeiten von Covid-19 mit Patientenbehandlung und der Patientenbehandlung unter Regelbedingungen. Sekundär wurden Selbst- und Fremdeinschätzung der simulierten Behandlung miteinander korreliert.

**Methodik:** 22 Examenskandidat*innen fertigten innerhalb von 4 Stunden jeweils eine mehrflächige adhäsive Front- und Seitenzahnrestauration an und führten eine endodontische Behandlung an einem Oberkieferprämolaren sowie ein parodontales Debridement eines Quadranten durch. Alle Behandlungen erfolgten an einem im Phantomkopf fixierten Modell. Einhaltung der vorgegeben Hygiene- und Abstandsrichtlinien waren ebenso Bestandteil der praktischen Prüfung wie eine Selbsteinschätzung der praktischen Leistungen. Jeder Prüfungsteil wurde von einem Prüfer anonym bewertet. Als historische Kontrolle dienten die Prüfungsergebnisse einer Kohorte aus dem Jahr 2019. Zur Auswertung beider Gruppen wurden Mittelwerte (Standardabweichung), non-parametrische Korrelationen (Spearman’s Rho) sowie Gruppenvergleiche (Mann-Whitney) berechnet.

**Ergebnisse: **Die praktischen Prüfungsleistung des Examens unter simulierten Behandlungsbedingungen waren mit Ausnahme der endodontischen Teilprüfung signifikant schlechter (P<0,05) als in der Kohorte, die ihre Staatsprüfung am Patienten absolvierte. Die Gesamtnoten in Kariologie und Parodontologie beider Gruppen, welche eine strukturierte theoretische Prüfung einbezieht, unterschieden sich nicht voneinander. Die Kandidat*innen bewerteten ihre Leistungen mehrheitlich schlechter als die Prüfer, eine Korrelation von Selbst- und Fremdeinschätzung war nicht gegeben.

**Schlussfolgerung:** Bei dem Vergleich von zwei Semestern resultierte eine simulierte praktische Prüfung ohne Patienten in den Fächern Kariologie, Endodontie und Parodontologie in vergleichbaren Resultaten wie eine Prüfung am Patienten. Gleiche Bedingungen für die Kandidat*innen, bessere Vergleichbarkeit und Vermeidung ethischer Dilemmata der Patientenbehandlung unter Prüfungsbedingungen könnten auch zukünftig Argumente für eine Staatsprüfung unter Phantombedingungen sein.

## Introduction

At the Polyclinic of Operative Dentistry and Periodontology of the Medical Faculty of the University of Cologne, an alternative examination model became necessary in March 2020 due to the acute COVID-19-related lockdown and the premise of enabling students to complete their state examination, which began in July 2019. The state examination should be carried out according to the guidelines of the North Rhine-Westphalian Ministry of Health and internal, university guidelines without patient contact and taking into account the required distance and hygiene guidelines. The implementation should be a simulation of a practical state examination in the subjects of restorative dentistry, endodontology and periodontology as realistically as possible and in accordance with the licensing regulations, while at the same time providing maximum protection for candidates, staff and examiners.

In addition to the organizational and content-related presentation of a procedure adapted to Covid-19 conditions it was the aim of the present retrospective study to compare examination results in the practical state examination in the subjects of cariology, endodontics and periodontology under simulated treatment conditions and patient treatment under standard conditions. Secondly, self-assessment of the candidates was correlated with the external assessment of the examiners in the simulated patient treatment.

## Methods

### Practical requirements in the COVID-19 exam

**Figure 1:**
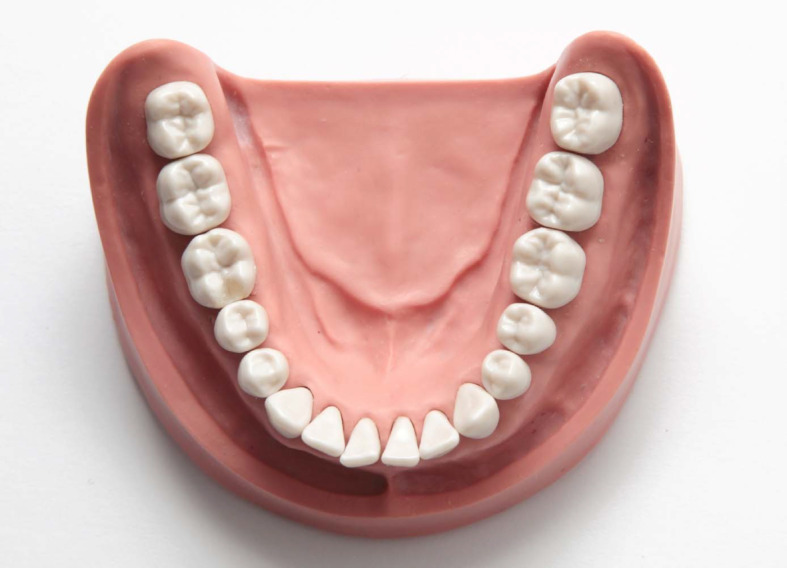
Exemplary sample of an adhesive posterior restoration of tooth 46

**Figure 2:**
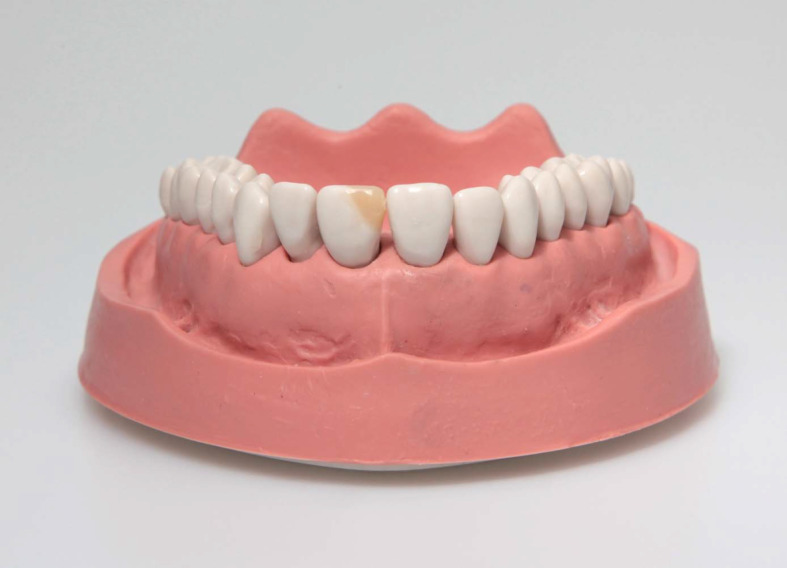
Exemplary sample of an adhesive incisal edge build-up of tooth 21

**Figure 3:**
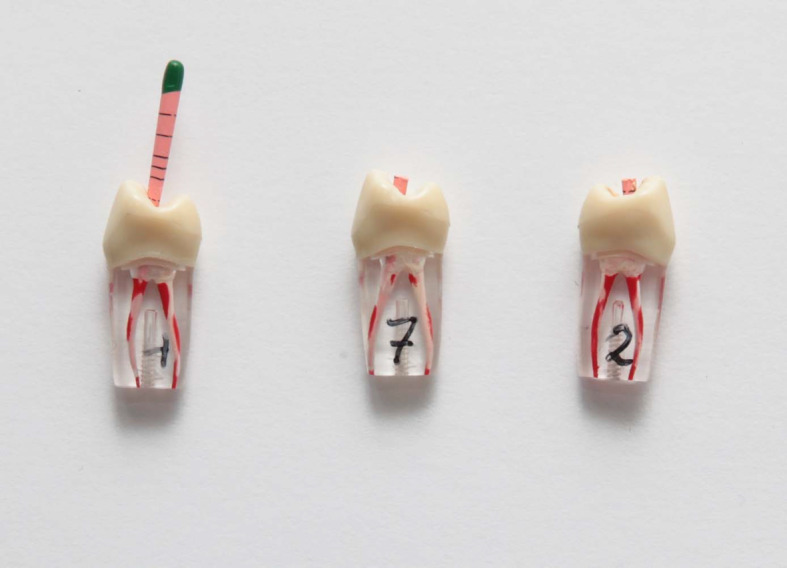
Three exemplary samples of endodontic treatments. During examination, the teeth were fixed in a model inside the phantom head

**Figure 4:**
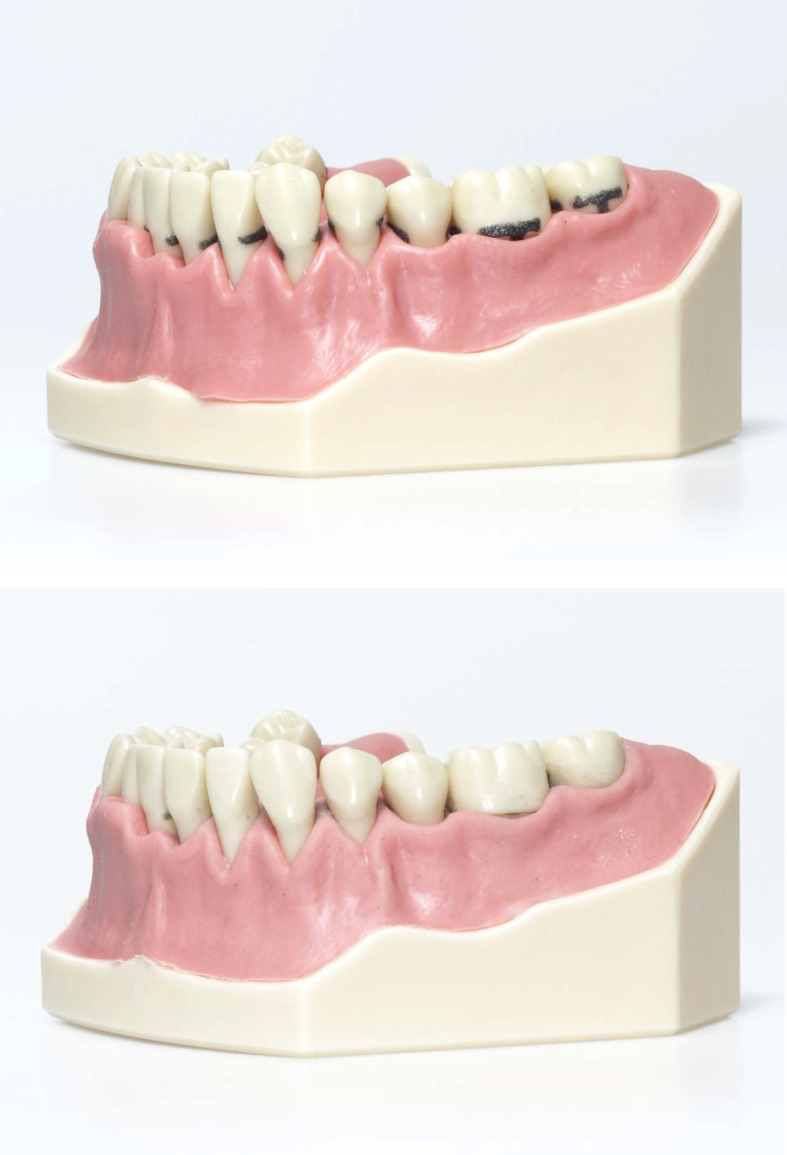
Model to simulate periodontal debridement before (a) and after depuration (b).

Based on the requirement profile of the old dental license to practice dentistry (ZÄPrO), the following examination tasks were defined:

1. a Class-II preparation on tooth 46 (see figure 1 (Fig. 1)) and a Class-IV preparation on tooth 21 (see figure 2 (Fig. 2)), including adhesive restoration in multi-layer technique;
2. trepanation, preparation and obturation of tooth 24 (see figure 3 (Fig. 3)) and
3. preparation of periodontal findings including classification (grading and staging) and risk assessment using a case vignette and periodontal debridement of the third quadrant, taking into account “patient positioning” and ergonomics (see figure 4 (Fig. 4)).

The examination tasks had to be completed within 4 hours; neither sequence nor time limit was specified for the individual partial services. For organizational reasons, the periodontological examination was started, since a model change was performed under supervision after completion. The detailed evaluation criteria of the partial performances are shown in table 1 (Tab. 1).

In addition to the practical tasks, individual theoretical examinations were conducted in the subjects of cariology (including restorative dentistry and endodontics), periodontology and pediatric dentistry.

#### Protection against infection and hygiene guidelines

The responsible hospital hygiene department and the study dean’s office approved conduct of the examination in advance. Candidates were allowed to participate in the trial only if they complied with the standard hygiene guidelines (distance rule, surgical mouthguard, safety goggles, disposable gloves) and without clinical COVID-19 symptoms, which was documented at the beginning of the exam together with the confirmation of eligibility.

**Figure 5:**
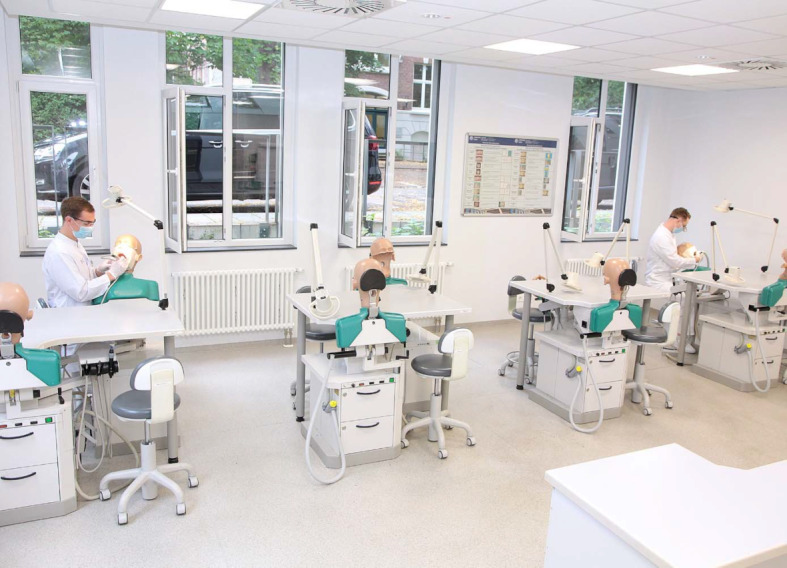
Simulation of the treatment situation in the course room with open windows and safety distance. The persons depicted were models and not examination candidates.

A logistics and distance plan for the course room as well as its access was planned beforehand, which allowed for minimum distances of the students to their places while observing the distance rules. The workplace was prepared at the beginning of the examination by the staff of the department, so that the candidates did not have to leave their place during the examination phase. The 22 participants were divided into two groups, which were spread over two examination rooms. The distance between the workstations was at least 3.5 m (see figure 5 (Fig. 5)). Self-disinfection and surface disinfection at the end of the examination as well as compliance with hygiene guidelines were additional components of the practical examination performance. 

#### Description of the exam candidates in the COVID-19 exam and historical control

Both the 22 COVID-19 exam candidates as well as the 26 candidates of a historical control group, who completed the state exam with patient contacts one year before, finished their dental studies before the outbreak of COVID-19 analogous to the exam regulations (ZÄPrO) of 1955. The students of the current year group had assisted the exam candidates in the historical control one year before.

#### Evaluation, data collection and data quality

In the exam with simulated patient treatment, an evaluation was carried out following the examination of all models or teeth pseudonymized by numbers by one examiner per subarea, whereas different examiners carried out the practical evaluations of the control group. 

In accordance with the procedure in the state examination with patient contact, the university grading scale in the gradations 1.0, 1.3, and 1.7 etc. to 5.0 was used to evaluate the model performance. After completing the practical work, the candidates documented their work on an evaluation sheet using the same grading scale.

For descriptive and comparative evaluation of both cohorts, mean values (standard deviation), non-parametric correlations (Spearman's Rho), and group comparisons (Mann-Whitney) were calculated. The anonymized, routine retrospective evaluation of the test results is ethically safe, as the presentation of the results does not allow for traceability. The local ethical board approved this retrospective study (dossier number 20-1470).

## Results

In the subjects of restorative dentistry and periodontology, the overall scores in the examination with phantom treatment did not differ significantly from the historical control from 2019. The scores for anterior and posterior restoration and periodontology were significantly worse under simulated model conditions and blinded assessment (see table 2 (Tab. 2)). With the exception of the posterior restoration, the self-assessment of the candidates was worse than the external assessment by the examiners in all other examination tasks, and there was no significant correlation between self-assessment and external assessment for any task.

## Discussion and conclusions

Although practical performance in periodontology and restorative dentistry was rated significantly worse in the simulation than on the patient, the overall scores did not differ in the two cohorts. This is mainly due to mathematical reasons, since in both forms of the examination a number of partial scores (practical and theoretical) are added and averaged, which are then rounded to give an overall assessment in whole grades. The significantly poorer assessment in the simulation could be because a more careful assessment after the examination was possible without the "patient" factor and secondly, the performance of the candidates could be compared. The detailed evaluation of practical performance is almost impossible on patients, but aspects such as patient management, but also anatomical or other patient-related challenges cannot be represented in a simulated examination, which could be a systematic distortion effect in the evaluation.

Both cohorts have undergone an identical clinical education and differ only in the way they are examined. They were two average semester sizes that did not differ in age and gender. The number of cases can only be seen as exemplary. Especially the ethical aspect of an examination on patients is controversially discussed: Advocates see the trial in patients as the logical conclusion of a clinical education, opponents see the lack of comparability and patient protection as ethical dilemmas that are not present in a model trial [1].

In the times of Covid-19, a state examination on model teeth could, at least in the final result, provide examination results that were almost identical to those of a cohort with patients. After the candidates have successfully completed two semesters of clinical teaching to provide evidence of patient treatment, this form of examination could also be discussed as a model for the future. From the point of view of infection control, there was no alternative in April 2020, but for ethical reasons and for better comparability, this type of state examination could also be an alternative in the future.

## Competing interests

The authors declare that they have no competing interests. 

## Figures and Tables

**Table 1:**
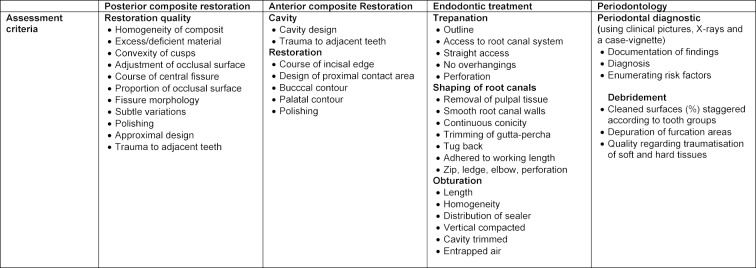
Assessment criteria for the simulated treatment for individual scenarios

**Table 2:**
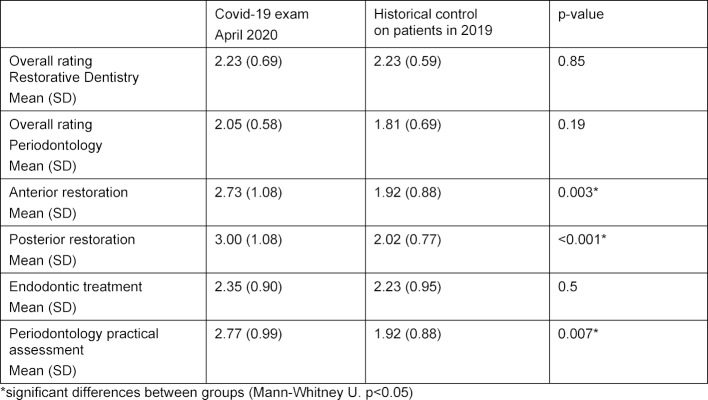
Partial and overall results of cohorts during Covid-19 and historical control on patients in 2019
